# “Pain Prehabilitation” in Major Joint Surgery: The Way Forward to Improve Outcomes and Prevent Pain Chronicity

**DOI:** 10.3390/jcm14217659

**Published:** 2025-10-28

**Authors:** Flaminia Coluzzi, Alberto Di Martino

**Affiliations:** 1 Department of Surgical and Medical Sciences and Translational Medicine, Sapienza University of Rome, 00189 Rome, Italy; 2 Unit Anaesthesia, Intensive Care and Pain Therapy, Sant’Andrea University Hospital, 00189 Rome, Italy; 3 1st Orthopedic Department, IRCCS—Istituto Ortopedico Rizzoli, 40136 Bologna, Italy; albertocorrado.dimartino@ior.it; 4 Department of Biomedical and Neuromotor Sciences—DIBINEM, University of Bologna, 40136 Bologna, Italy

**Keywords:** arthroplasty, osteoarthritis, post-operative pain, chronic post-surgical pain, prehabilitation, peri-operative pain, knee, hip

## Abstract

Osteoarthritis (OA) is one of the most frequent orthopedic disorders and a common cause of chronic pain, which is one of the most important factors in recommending total joint arthroplasty (TJA). Due to a greater need for pain relief and improved mobility in the OA population, TJA procedures are in high demand, and most patients with OA experience long waiting times. Waiting for TJA places a significant burden on patients as a result of worsening pain and functional deterioration. Therefore, optimizing pre-operative circumstances in these patients is essential to target analgesic interventions, preserve post-operative quality of life, and minimize post-operative outcomes such as chronic post-surgical pain. Achieving optimal pain control before surgery remains an unmet need, and it is difficult to devise a one-size-fits-all analgesic regimen. Pain is a challenge for orthopedic healthcare professionals (OHCPs), and orthopedic patients are notably less satisfied than patients undergoing other surgeries in terms of pain management. We reviewed the latest clinical evidence on pain management in patients with OA wait-listed for TJA to help OHCPs effectively manage their pain. Here, we provide actionable suggestions to strengthen orthopedic surgeons’ competency in pain assessment and therapy selection. By integrating the perspectives of an orthopedic surgeon and a pain therapist, we also introduce the concept of “pain prehabilitation” and propose integrating it into standard care protocols during the TJA wait-list period to optimize TJA outcomes and prevent the development of chronic post-surgical pain.

## 1. Introduction

Osteoarthritis (OA) is a leading cause of disability worldwide, affecting almost 600 million individuals in 2020 [1,2,3,4,5]. By 2050, cases of knee and hip OA are expected to increase by >70%, thus placing a significant toll on healthcare systems [2]. Moderate to severe pain occurs across the OA disease continuum and along the entire patient journey from OA diagnosis to surgical intervention. Pain has a severe impact on patient satisfaction; almost one in two patients with OA feel dissatisfied with the prescribed pain-relieving interventions [6,7,8]. Studies assessing the patterns of prescription habits among clinicians managing patients with OA in EU countries suggest that non-steroidal anti-inflammatory drugs (NSAIDs) are widely prescribed at a higher percentage compared with opioids and intra-articular injections (54.6% in Italy; 50.87% in Ireland; 27% in the United Kingdom; 44.1% in Germany) [9,10,11,12] than in the United States, where opioids were historically more commonly prescribed [13,14], despite a recent trend of decreased use in favor of NSAIDs [15,16,17].

Pain is a leading reason why patients seek referral to an orthopedic surgeon [5,18] and one of the most important factors in recommending total joint arthroplasty (TJA). Due to a greater need for pain relief and improved mobility in the OA population, TJA procedures are in high demand [19,20,21], and most patients with OA can experience long waiting times when accessing the National Health System. On average, according to the Organisation for Economic Co-operation and Development (OECD), in 2020, patients with OA wait-listed for elective THA and TKA waited about 113 days and 189 days, respectively, before being admitted to hospital [22]. Waiting for TJA places a significant toll on patients because they experience worsening pain, functional limitations, and loss of health-related quality of life [23], with more significant deterioration in patients with knee OA compared with those with hip OA [24]. Moreover, patients awaiting surgery for more than 6 months have been shown to be at increased risk for poor outcomes after TJA [25]. Although TJA is an effective treatment in most people with end-stage OA, 20–40% of patients feel dissatisfied, experience chronic post-surgical pain (CPSP) [26], and report residual physical limitations even 6–12 months after surgery [27].

Pre-existing chronic pain is the strongest predictor of poor acute post-operative pain control, along with anxiety and age [28]. Pre-operative chronic pain is also a strong factor in predicting the risk of developing CPSP [29,30,31,32]. Inadequately treated acute post-operative pain adversely affects recovery by increasing the risk of complications, delaying discharge and return to function [29,33]. Therefore, interventions aimed at effective pain management in wait-listed patients with OA have the potential to facilitate patient prehabilitation before surgery and to improve post-surgical outcomes, while minimizing the risk of post-operative complications, including CPSP.

Pain is a challenge for orthopedic healthcare professionals (OHCPs) [5], and orthopedic patients are notably less satisfied than patients undergoing other surgeries in terms of pain management [34]. International guidelines [35,36,37,38,39,40] provide little guidance on pain therapy selection before TJA. The available evidence stems mostly from single-center studies or consensus documents. We have reviewed the latest clinical evidence on pain management in patients with OA wait-listed for TJA and provided actionable suggestions to strengthen orthopedic surgeons’ competency in pain assessment and therapy selection. By integrating the perspectives of an orthopedic surgeon and a pain therapist, we have introduced for the first time the concept of “pain prehabilitation” as a promising process of pre-operative pain management, with the key benefit of optimizing TJA outcomes and preventing the development of CPSP. Pain prehabilitation is thought to be analogous to what has been already proposed for other settings of pre-operative optimization, such as exercise, nutrition, mental health, and lifestyle changes. For the purpose of this paper, we define pain prehabilitation as outpatient pre-operative interventions aimed at optimizing the control of OA-associated pain to promote functional recovery and prevent pain chronicity.

## 2. Methods

Based on their proficiency in managing pain in patients with OA who are eligible for TJA, as well as their publications in these domains, F.C. and A.D.M. first posed the following questions: (a) What clinical profile is most encountered among patients with OA awaiting TJA? (b) In patients with OA who are eligible for arthroplasty, could the identification of predictive factors of unsatisfactory pain control after surgery help OHCPs optimize outcomes? (c) Would specific “pain prehabilitation” protocols help OHCPs to adequately manage pain in patients undergoing elective TJAs?

The authors then conducted a comprehensive, non-systematic review of the existing literature on three main scientific databases, namely, PubMed/MEDLINE, Scopus, and Web of Science, to address these questions. Articles for consideration were retrieved using different combinations of keywords (e.g., osteoarthritis AND arthroplasty AND pain management AND waitlist AND chronic post-surgical pain) and were selected for inclusion if judged relevant to the topic. Between January and March 2025, the authors had several meetings to review the literature findings and develop expert opinion-based insights.

## 3. What Clinical Profile Is Most Encountered Among Patients with OA Awaiting TJA?

Pain is the dominant symptom in patients with OA [41], and for many, recurring changes in the intensity and duration of pain underlie the lived experience of OA and drive primary healthcare consultations [42]. Initially, pain in OA joints is nociceptive with sporadic occurrence on movement and on joint loading, and specific activities may trigger this pain [43]. At advanced stages of OA, patients show signs of central sensitization, including lower pressure pain thresholds and higher pain summation scores on repetitive stimulation, which may contribute to pain severity [41,44]. The presence of widespread pain sensitization before surgery may explain why the removal of the peripheral pain source after TJA may not eliminate augmented central pain-processing changes [45]. Overall, patients with OA at an advanced stage experience severe pain, complete loss of organ reserve, and synovial joint failure, which contribute to patient frailty [42,46].

Patients with OA who fulfil the eligibility criteria for TJA are mostly >60 years of age (mean age at surgery, 64–71 years and 66–72 years for hip and knee arthroplasty, respectively; the proportion of women, range 52–67% for THA, 57–80% for TKA) and have primary OA (87–96%) [19]. Patients with OA are almost two times more likely to have multi-morbidities than their peers without OA [47], and patients with hip OA are slightly less likely to have other comorbidities than those with knee OA [48]. Hypertension, heart disease, type 2 diabetes mellitus (T2DM), and lung disease are the most frequent comorbidities encountered among patients with OA. Anxiety and depression are also prevalent in approximately one in five patients with OA [48]. As the number of comorbidities increases, so does the pain intensity and the use of pain medications [48]; a high number of comorbidities is widely recognized as a common predictor of deterioration in pain and physical functioning in patients with knee and hip OA. Increased prescription of NSAIDs has been documented among patients with OA [49] since diagnosis. However, the efficacy of NSAIDs in relieving pain and improving function usually wanes over time [50], and long-term use of NSAIDs was found to make progression to TKA faster by significantly aggravating symptoms [51]. A recent longitudinal study revealed that individuals with knee OA on long-term use of NSAIDs over 4–5 years may experience worsening of symptoms (including pain, disability, and stiffness scores) that exceeds the minimally clinically important difference, a parameter that defines what is clearly perceived by the patient as either beneficial or harmful [51]. Moreover, NSAIDs are not recommended in elderly patients, especially, but not only, because of possible impairment of renal function, and they are strongly contraindicated in patients with renal failure [52].

In the routine orthopedic setting, specific comorbidities can preclude the patients from undergoing surgery; these include acute myocardial infarction, cerebrovascular disease, and malignant tumors. In contrast, end-stage renal disease requiring hemodialysis, liver cirrhosis (Child–Pugh C), uncontrolled congestive heart failure, and severe chronic obstructive pulmonary disease may be regarded as modifiable contraindications for elective TKA or THA [53]. However, pending appropriate evaluation and optimization of the risk factors for complications to develop (e.g., deep vein thrombosis treatment via anticoagulant therapy for at least 3–6 months), patients can then be re-evaluated and, if regarded eligible by the anesthesiologist, may undergo surgery. However, even patients with advanced OA who are found to be eligible for TJA by the orthopedic surgeon may be unwilling to consider surgery [54,55]. In an observational study involving >30,000 individuals with knee or hip OA, the proportion of patients willing to undergo surgery was higher in the presence of more severe symptoms and disability; worsening pain was the reason to reconsider surgery within the following 3–12 months among those who were unwilling initially [56,57].

Overall, moderate to severe pain associated with OA drives medical consultations and willingness to undergo surgery. While on the TJA wait-list, pain should be appropriately controlled, considering that patients with OA are often dissatisfied with the prescribed pain-relieving medications [8,58,59]. In this scenario, predicting which patients are more prone to CPSP after TJA surgery may promote the adoption of effective interventions while on the surgical waiting list that may ultimately optimize outcomes, including the risk of chronification.

## 4. In Patients with OA Who Are Eligible for Arthroplasty, Could the Identification of Predictive Factors of Unsatisfactory Pain Control After Surgery Help OHCPs Optimize Outcomes?

Pre-operative identification of high-risk patients regarding post-operative complications is paramount in orthopedic surgery because complications pose a significant psycho-social burden on the patient and substantial costs to the healthcare system. Mounting evidence suggests that pain experience itself is one of the most critical predictors of pain deterioration, and pre- and post-operative pain are significant risk factors [60]. With regard to psychological characteristics, pain catastrophizing, anxiety, and depression are frequently highlighted as predictors of CPSP after TJA [60].

In patients with knee OA, female gender, lack of multimodal analgesia, unrelieved pain, and impaired function were associated with the occurrence of CPSP [61]. In patients with hip OA, pre-operative pain, pre-operative depression, and type of analgesic were found to be independent risk factors for CPSP [62]. In patients waiting for TJA, the more severe the pain, the slower the recovery, the more delayed the discharge, and the more intense CPSP will be [63].

Patients with high levels of pre-operative pain are more likely to report CPSP after TJA [64]. The association between pre-operative pain and CPSP measured 1 year after surgery is significant in patients undergoing both TKA and THA [64,65]. Therefore, implementing pain management strategies pre-operatively may prove effective and beneficial for patients with OA, particularly if targeting pain-at-rest and pain-on-movement in patients undergoing THA and TKA, respectively [64].

Frailty, a syndrome characterized by limited physiological reserve and ability to cope with surgery-related distress, is one of the main factors to consider for risk classification and outcome estimation. In routine practice, patients can be assessed for frailty using the FRAIL Scale, which is based on five items (fatigue, resistance, ambulation, illnesses, and loss of weight), easily extracted during patient history taking and examination [66]. Patients undergoing TJA who reported at least three of five items of the FRAIL Scale experienced higher rates of complications after TKA and THA [67], including CPSP. Pre-operative frailty was correlated with post-surgical complications in patients undergoing THA, and this was significantly associated with the occurrence of CPSP [68,69]. In patients with knee OA, pain is a major contributor to the development of frailty; these patients experience severe pain and are significantly more likely to have frailty compared with their counterparts without pain [70]. In addition, pre-operative medications were found to influence outcomes after TJA; therefore, managing complex anamnesis makes elective TJA patient care challenging. In line with this, patients taking more medications were more frequently discharged to an extended care facility and had increased length of stay and re-admission rates [71]. The category and quantity of pre-operative medications can predict outcomes after TJA; anti-diabetics and narcotics (e.g., fentanyl and methadone) have the most significant influence. The medications that patients should stop taking before surgery to minimize post-surgery complications are listed in Table 1 [72,73,74,75,76,77,78,79,80,81].

Overall, pre-operative pain is not only a relevant predictor of CPSP and worse post-surgical outcomes, but it also contributes to patient frailty, which correlates directly with post-surgery complications. Therefore, as advocated by de Ladoucette, pain should be managed as early as possible in the pre-operative period and limited for as long as possible in the post-operative period to avoid it becoming chronic [63]. In the last decades, Enhanced Recovery After Surgery (ERAS) protocols have been widely introduced in clinical practice; these have been shown to significantly affect clinical outcomes in terms of length of hospital stay, morbidity, and mortality. In 2020, specific ERAS recommendations were issued for TKA and THA [82]. These recommendations in the pre-operative period, aiming for patient optimization before admission, include smoking and alcohol cessation, correction of anemia, pre-operative physiotherapy, and guidelines on pre-operative fasting. Surprisingly, despite increasing evidence of the relevant role of pre-existing chronic pain as a risk factor for CPSP, recent guidelines do not include analgesia optimization in current prehabilitation programs. Current prehabilitation protocols mostly consist of one or more pre-operative interventions, such as exercise, nutrition, psychological strategies, patient education, and respiratory training, aimed at enhancing functional and metabolic capacity and physiological reserve to allow patients to withstand surgical stressors, improve post-operative outcomes, and promote recovery [83]. In this context, conditioning the body in terms of pain optimization may serve as a complementary approach to be adopted during the TJA wait-list period. Therefore, we propose introducing the concept of “pain prehabilitation” as a promising means to optimize TJA outcomes and prevent the development of CPSP.

## 5. Would Specific “Pain Prehabilitation” Protocols Help OHCPs to Adequately Manage Pain in Patients Undergoing Elective TJAs?

Recent advances have shed light on pain pathways in OA, showing that although joint damage and inflammation are the main causes of pain generation, central and peripheral nervous system mechanisms aggravate symptoms and contribute to pain chronification and disability [44]. In advanced disease, neuropathic pain can be present as well as signs of central sensitization, which collectively contribute to the transition to chronic pain and enhanced pain intensity [84,85]. To date, persistence of central sensitization with an apparent lack of peripheral input might explain why some patients experience CPSP after a technically successful TJA [84].

A mechanism-based approach to OA-associated pain [86] should target the peripheral mechanisms of inflammation, the central mechanisms of pain sensitization, and prevention of joint degeneration (peripheral pain generators) [44,87]. In OA, two primary peripheral sources of pain have been identified, namely, subchondral bone pressure and inflammation. Subchondral bone pressure, as a result of cartilage damage and subchondral oedema, has been found to be more associated with pain at rest, which stands as the most relevant pain mechanism in hip OA. In contrast, inflammation, associated with pain-on-movement, seems to be a relevant contributor in knee OA [32,64]. Nevertheless, the waiting period before surgery is characterized by mixed chronic pain, given the coexistence of both nociceptive and neuropathic components [88].

Appropriate selection of pain therapy requires understanding of the underlying analgesic mechanism of action of the prescribed medications. By switching off peripheral sensitization, NSAIDs are effective for acute pain when inflammation is the driving force of nociception; therefore, their use as first-line therapy aimed at treating inflammatory nociceptive pain can be appropriate. Conversely, when pain shows signs of progression and patients experience central sensitization, the use of NSAIDs is no longer appropriate because other mechanisms are involved in pain perception, including spinal sensitization and underlying maladaptive neuronal plasticity, leading to pain becoming a chronic disease [89]. Moreover, the feasibility of NSAID therapy relies on the patient’s status because the use of NSAIDs is hindered by older age, comorbidities, and polypharmacy [90]. NSAIDs are not recommended for prolonged treatment because of the risk of cardiovascular, gastrointestinal, and renal toxicities, especially with long-term use [52,87]. Furthermore, individuals on long-term NSAIDs (4–5 years) are significantly more likely to experience aggravated symptoms, which can accelerate progression to total joint replacement compared with non-users [51]. The use of NSAIDS in patients waiting for TJA has also been associated with a twofold increase in bleeding after THA [76] as well as increased frailty [91], including the risk of deterioration of renal function [52,92]. Despite this, NSAIDs are widely prescribed, regardless of the physician specialty, with usage ranging from 55% to 27% in most European countries [8,10,11,12] and for a time that mostly exceeds what is defined by their label.

Pain chronification requires central analgesics to target the amplification of ascending pain signals and inadequate activation of descending inhibitory signals because of pro-nociceptive neuroplasticity [93]. The use of oral opioids in patients on the waiting list for TJA has been recommended as the last pharmacological option before surgery by national [35] and international guidelines [36]. In addition, no consensus exists regarding the association of pre-operative opioid use with post-operative complications. Evidence from patients in the United States suggests that chronic use of opioids pre-operatively is associated with increased post-operative use, enhanced surgical site infections, and greater risk of a revision procedure [77,78,79]. In contrast, a retrospective study carried out in Australia showed that pre-operative opioid use was not associated with post-operative adverse events, including pain [80]. Not all opioids used in clinical practice are the same [94]; most of the commonly used opioids act as full agonists at μ-opioid receptors (MORs), with individual differences in affinity and efficacy at δ-opioid receptors and κ-opioid receptors, thereby producing pain relief and undesired effects through a single opioid mechanism [95]. However, some compounds, often referred to as “atypical” or “multi-mechanistic” opioids, have analgesic properties targeting both ascending opioid pathways and non-opioid descending pathways (e.g., tramadol, buprenorphine, and tapentadol). For chronic OA pain, descending inhibitory pain pathways are often disrupted, and agents targeting one of these, such as norepinephrine reuptake, may be more appropriate for managing chronic pain than pure MOR agonists [87,88]. In patients with uncontrolled OA-associated pain who were waiting for TJA, a short-term treatment with tapentadol, the archetype of the class of compounds named MOR-NRIs (norepinephrine reuptake), was found to provide meaningful analgesic benefits and lower incidence of gastrointestinal adverse events compared to oxycodone [96]. In addition, compared to other medications commonly employed in patients with OA, tapentadol displays the advantage of not requiring CYP enzyme activation (e.g., over tramadol) and no interference with blood clotting [e.g., over cyclo-oxygenase (COX) inhibitors]. Overall, such features may be relevant while waiting for surgery because there is no need to discontinue pain therapy, as required with the use of NSAIDs [97].

## 6. Expert Opinion

In the next few decades, there will be an increase in the demand for primary arthroplasties and revision procedures [98,99], resulting in more patients experiencing long wait times before surgery [100]. Waiting for TJA places a significant burden on patients, with worsening pain and functional deterioration; therefore, optimizing pre-operative circumstances in these patients is essential to target analgesic interventions, preserve post-operative quality of life, and minimize post-operative outcomes such as CPSP [101]. As a multidimensional sensory experience, pain demands a multidimensional and multidisciplinary approach to its control based on the biopsychosocial model of pain [102], which implies interventions within holistic and multidisciplinary frameworks for treating pain. Therefore, the management of a painful TJA should be entrusted to a multidisciplinary team involving orthopedic surgeons, physical therapists, pain management physicians, and primary medical doctors [103,104]. A full assessment of surgical and non-surgical factors that can cause pain after TJA should be performed irrespective of the origin of the pain and whether it can be addressed surgically [105]. During the wait-list period, a pre-operative risk assessment of CPSP is of paramount relevance [29]; however, an individualized quantitative tool to predict the probability of CPSP accurately and effectively in orthopedic patients is lacking [106]. In this scenario, pre-operative scores such as the Amsterdam Preoperative Anxiety and Information Scale (APAIS) and DN4 neuropathic pain diagnostic score have been documented to effectively predict CPSP [63], and these can easily be used in routine practice. Recent evidence indicates that between hospital discharge and the proposed CPSP cut-off (10 days to 3 months after surgery), a pain condition known as transitional pain occurs and has significant potential in predicting the development of CPSP. To this end, a holistic approach involving the establishment of a “transitional pain service” has been recently recommended by the European Society of Regional Anaesthesia and Pain Therapy (ESRA) as a promising strategy to facilitate CPSP prediction and mitigate its burden [107].

**Figure 1:**
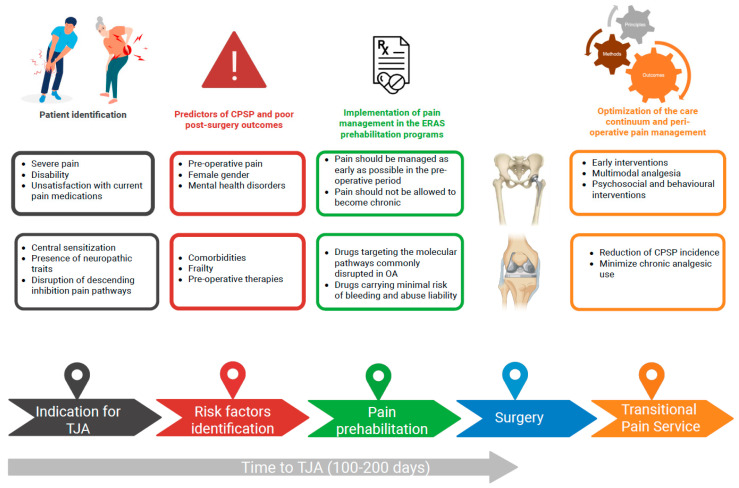
Management of osteoarthritis during the total joint arthroplasty wait-list period: focus on pain. CPSP, chronic post-surgical pain; TJA, total joint arthroplasty. Created in BioRender. https://BioRender.com/jashqay (accessed on 20th October 2025).

Achieving optimal pain control before surgery remains an unmet need, and it is difficult to devise a one-size-fits-all analgesic regimen [84]. Nevertheless, interventions aimed at providing effective pain control in wait-listed patients with OA are promising in terms of facilitating patient prehabilitation to surgery, improving post-surgery outcomes, and minimizing the risk of post-operative complications, including CPSP (Figure 1).

However, guidelines regarding appropriate pain treatment during the TJA wait-list period are still lacking, and the current concept of prehabilitation does not include pain optimization. Even in contemporary prehabilitation programs, neither specific recommendations nor mechanism-based indications on pain therapies are issued for pain control before surgery, including preference of one medication over another [35,36,37,38,39,40]. In this context, our proposal of integrating the “pain prehabilitation” concept aims to raise awareness of the impact of inadequate control of pain in patients wait-listed for TJA on post-surgery outcomes. Moreover, pending clinical evidence from studies assessing “pain prehabilitation” in routine orthopedic practice, our proposal may inform management approaches and guide treatment choices.

Implementing pain prehabilitation in routine practice can be challenging due to resource allocation; the need for interdisciplinary coordination between anesthesiologists, pain therapists, and surgeons; and patient’s adherence to pre-operative interventions. To this end, educational initiatives targeting both clinicians and patients with OA that aim to optimize pain levels before surgery to prevent chronicity could be helpful. Moreover, pending additional evidence of effectiveness of pain prehabilitation in the pre-operative management of patients awaiting TJA, potential integration of pain prehabilitation in ERAS protocols may facilitate its implementation in real-world practice.

Despite advances in our understanding of specific mechanisms of pain, the most frequently prescribed pain treatments lag far behind our knowledge, with a significant proportion of patients with advanced stage OA taking pain medications that do not target the underlying pain mechanisms appropriately across OA-associated pain patterns over time [108]. To date, one in four patients have been taking NSAIDs, with their use increasing substantially (one in three patients) as the time of surgery approaches. The use of opioids significantly increases during the pre-surgery period, suggesting the presence of relatively high levels of pre-operative pain not adequately controlled by previous pharmacological trials. To this end, pain-relieving medications that target the pain mechanisms characterizing the course of OA pain and contribute to its chronification, including both ascending opioid pathways and non-opioid descending pathways, may hold greater potential to manage pain effectively during the wait-list period. Interventions preventing pain chronification may be more successful if a tailored assessment of vulnerability factors can be obtained [60]. In this scenario, frailty assessment should be implemented incrementally in routine patient preparation for TJA to minimize the risk of CPSP. Furthermore, it is also important to manage analgesics in the pre-operative period within the context of ERAS protocols, and “pain prehabilitation” should be integrated in such routine practice along with educational interventions to optimize patients’ ability to manage and cope with pain. Pain catastrophizing, kinesiophobia, and ineffective pain-coping methods affect patients’ physical capabilities and can impede the effectiveness of pain management and interventions. Managing patient expectations of pain associated with TJA remains critically important [6]; unmet pre-operative expectations lead to patient dissatisfaction post-operatively [109]. To this end, at every pre-operative evaluation, clinicians should talk with patients about their desires and expectations because these factors play a key role in predicting outcomes and satisfaction after elective orthopedic surgery.

## 7. Conclusions

The integration of “pain prehabilitation” into standard care protocols during the TJA wait-list period can improve patient outcomes. Therefore, a pivotal role for orthopedics is emerging in OA management from both surgical and non-surgical perspectives, with greater demand for their commitment to pain management. Actionable suggestions are presented in Table 2 to strengthen competency in pain assessment and therapy selection during the TJA wait-list period.

A multidisciplinary approach, with referral to pain therapists, may be necessary in specific conditions. The more effective pain control is, the more patients can recover, and the more satisfactory the functional outcome will be [63].

## Figures and Tables

**Table 1:** Commonly used medications among patients with OA: considerations for evaluating the need for suspension.

Medication	Suspension Regimen	Notes	References
Anti-diabetics	SGLT-2 inhibitors: 3 days Metformin, GLP-1RA analogues, and DPP-4 inhibitors can be continued, including on the day of surgery	SGLT-2 inhibitors have been reported to be associated with euglycemic ketoacidosis (euDKA), and surgery may aggravate euDKA as the surgical stress response increases ketone production	[71,72]
Aspirin, clopidogrel, warfarin	Aspirin is usually continued Clopidogrel: 5–7 days Warfarin: 3–5 days	Once the surgical hemorrhagic risk has been defined, it is advisable to carefully evaluate each patient’s risk and balance it with the thrombotic risk	[73,74]
DOAC	DOAC: 2 days	Pre-operative interruption and post-operative DOAC resumption are necessary to minimize the risks of thromboembolism and bleeding	[74,75]
NSAIDs	NSAIDs: 7–10 days	Non-selective NSAID therapy is associated with a twofold increase in bleeding after total joint arthroplasty	[76]
Opioids (codeine, tramadol, buprenorphine, tapentadol, morphine, fentanyl, oxycodone, hydrocodone, hydromorphone, meperidine)	Opioids are usually continued or switched to IV formulations	Although chronic use of opioids pre-operatively has been linked to increased consumption after surgery, along with increased surgical site infections and revision risk, in patients taking opioids for chronic pain, it is advisable to guarantee baseline requirements of opioids, regardless of the anesthetic technique. Do not induce weaning during the peri-operative pain management	[77,78,79,80,81]

GLP-1RA, glucagon-like peptide-1 receptor; DPP-4, dipeptidyl peptidase 4; SGLT-2, sodium-glucose transport protein 2; DOAC, direct oral anticoagulant; NSAIDs, non-steroidal anti-inflammatory drugs.

**Table 2:** Actionable suggestions from the experts.

Orthopedic surgeons should carefully assess the patient’s main complaint, namely pain underlying disease, and identify red flags before the surgery to minimize unsatisfactory outcomes post-operatively
Understanding the trajectories of patient pain status while on the wait-list could aid clinicians in assessing the risk of deterioration of patient function and poor outcomes after TJA
Identification of signs of central sensitization may be of help in prescribing the most appropriate analgesic therapy during the TJA wait-list period
Pain-relieving medications that target the pain mechanisms characterizing the course of OA pain and contribute to its chronification, including both ascending opioid pathways and non-opioid descending pathways, may have greater potential to manage pain effectively during the TJA wait-list period
Female patients with comorbidities, high psychological distress, and uncontrolled pain despite treatment should be promptly identified and enrolled on pain prehabilitation programs while they wait
A pre-operative risk assessment of CPSP is of paramount relevance and can be obtained in collaboration with a pain therapist with the final aim of improving surgery outcomes and minimizing surgery complications
Early identification of individuals with CPSP is an important component of intervention, because addressing pain in a timely manner is likely to reduce the risk of long-term persistence
During the shared decision-making process, the patient’s individual expectations should be discussed, and their actual feasibility by means of TJA should be evaluated

TJA, total joint arthroplasty; OA, osteoarthritis; CPSP, chronic post-surgical pain.

## Data Availability

Not applicable.

## Abbreviations

The following abbreviations are used in this manuscript:

APAIS	Amsterdam Preoperative Anxiety and Information Scale
COX	Cyclo-oxygenase
CPSP	Chronic post-surgical pain
DOAC	Direct oral anticoagulant
DPP-4	Dipeptidyl peptidase 4
DKA	Diabetic keto-acidosis
ERAS	Enhanced Recovery After Surgery
ESRA	European Society of Regional Anaesthesia and Pain Therapy
GLP-1 RA	Glucagon-like peptide-1 receptor agonist
NSAIDs	Non-steroidal anti-inflammatory drugs
OA	Osteoarthritis
OECD	Organisation for Economic Co-operation and Development
OHCP	Orthopedic healthcare professional
SGLT-2	Sodium-glucose transport protein 2
THA	Total hip arthroplasty
TKA	Total knee arthroplasty
TJA	Total joint arthroplasty
T2DM	Type 2 diabetes mellitus
